# Health Status of Visitors and Temporary Residents, United States

**DOI:** 10.3201/eid1511.090938

**Published:** 2009-11

**Authors:** Emad A. Yanni, Nina Marano, William M. Stauffer, Elizabeth D. Barnett, Maria Cano, Martin S. Cetron

**Affiliations:** Centers for Disease Control and Prevention, Atlanta, Georgia, USA (E.A. Yanni, N. Marano, W.M. Stauffer, M. Cano, M.S. Cetron); University of Minnesota, Minneapolis, Minnesota, USA (W.M. Stauffer); Boston Medical Center, Boston, Massachusetts, USA (E.D. Barnett)

**Keywords:** Health status, tuberculosis and other mycobacteria, measles, poliomyelitis, vaccine preventable diseases, temporary residents, United States, perspective

## Abstract

Human mobility has always been associated with the spread of infection, and mobility of nonimmigrant visitors and temporary residents to the United States is increasing, from ≈12 million in 1987 to ≈37 million in 2007. Lack of information about the health status of these populations upon arrival and their need for and use of medical services in the United States hinders development of public health policy, education, and provision of adequate clinical care. After these issues and needs are clarified, intervention programs should be developed to increase access and decrease the disparities of care experienced by these populations.

## Abstract 2

Education, policy, and interventions to promote visitor health are needed.

Each year, millions of nonimmigrants visit the United States. Nonimmigrants are defined by the Department of Homeland Security as foreign nationals granted temporary admission into the United States for a specific purpose (e.g., business, pleasure, academic or vocational study, or temporary employment) or to act as a representative of a foreign government or international organization (*1*). The number of nonimmigrant visitors and temporary residents to the United States increased from ≈12 million in 1987 to ≈37 million in 2007.

Human mobility has always been associated with the spread of infections such as smallpox or dengue fever. Increased speed of transportation accompanied by an exploding human population has created a situation ripe for the spread of infectious diseases. This spread of human pathogens may be manifested through an epidemic or pandemic (e.g., influenza A pandemic [H1N1] 2009 virus, HIV/AIDS, severe acute respiratory syndrome), introduction of a pathogen (e.g., West Nile virus, chikungunya virus) into a new or reestablished ecologic niche, or the spread of organisms that carry resistance or mechanisms of resistance to antimicrobial drugs (*2*). As with all mobile populations, visitors and temporary residents to the United States may represent a risk for public health through introduction of infections or vaccine-preventable diseases. Influenza A pandemic (H1N1) 2009 virus, responsible for the recent outbreak in Mexico, was subsequently transmitted across the borders to other countries, largely by returning US travelers but also through nonimmigrant visitors from Mexico (*3*). Of 178 pandemic (H1N1) 2009 patients for whom travel histories were available, 145 (82%) reported recent travel to Mexico and 4 (2%) reported travel to the United States. Among those who had not traveled to Mexico, 17 (52%) reported contact with a returning traveler from Mexico. Canada, Germany, Spain, and the United Kingdom all have reported evidence of in-country, second-generation, human-to-human virus transmission (*3*).

Although each year millions of visitors and temporary residents visit the United States, little is known about the health status of these populations. Some published reports provide a glimpse of the effects of infectious and chronic diseases carried by arriving immigrant populations, but few reports and no summarized data specifically address how visitors and temporary residents to the United States are affected by health risks such as trauma and injuries, chronic diseases, and infectious illness (*4*–*7*). Lack of information hinders development of public health policy, education, and provision of adequate clinical care for visitors and temporary residents. Therefore, to raise awareness of this traveling population, we have summarized the sparse available literature and call for future education, policies, and interventions geared toward promoting health and well-being for the visitors and temporary residents and public health protection to host communities.

## Description of Visitors and Temporary Residents to the United States

During 2007, a total of 171 million nonimmigrants were admitted to the United States: 134 million (78%) were Canadian and Mexican travelers who acquired border-crossing cards for the purpose of tourism or business, and 37 million (22%) were travelers with I-94 forms (applications required for nonimmigrant admission to the United States; Mexican nationals with border-crossing cards and tourists and business travelers from Canada are generally exempt from the I-94 requirement) (*1*). Most persons admitted with I-94 forms were temporary visitors such as tourists and business travelers (33.3 million; 89%), short-term residents (3.6 million; 10%), and expected long-term residents (205,000; 1%). Those admitted as temporary residents included short-term residents (e.g., temporary workers and families, students, exchange visitors [students participating in an exchange program], diplomats) and long-term residents (e.g., alien fiancés and spouses of US citizens or permanent residents and their children). Nonimmigrant admission refers to the number of events (entries into the United States) rather than persons. In 2007, the 10 countries with the most I-94 form admissions to the United States were India (11%), Mexico (11%), Japan (7.5%), United Kingdom (6.3%), South Korea (6%), Canada (6%), Germany (4%), People’s Republic of China (4%), France (3%), and Brazil (2%). From 2006 through 2007, the largest increases in resident nonimmigrant admissions came from citizens of Mexico (36% increase), India (30% increase), and China (27% increase), largely accounted for by increased numbers of seasonal workers, academic students, workers in specialty occupations, and intracompany transferees (*1*). From 2005 through 2007, the 10 most common destination states were California (14%), New York (13%), Texas (8.2%), Florida (7.7%), New Jersey (4.4%), Massachusetts (4%), Illinois (3.4%), Virginia (3.2%), Michigan (2.8%), and Pennsylvania (2.7%). The first 5 states represented the declared destinations of nearly 50% of the foreign nationals admitted in 2007 (*1*).

A study by the Office of Immigration Statistics estimated that during 2004, on any typical day, 3.8 million visitors and temporary residents were in the United States: 2.3 million (61%) tourist and business travelers, 704,000 (18%) temporary workers, and 640,000 (17%) students and exchange visitors (*8*). The mean lengths of visit were as follows: tourists and business travelers, 22 days; diplomats, 13 weeks; temporary workers, 23 weeks; students and exchange visitors, 31 weeks; and long-term residents, 43 weeks (*8*).

## Health Regulations for Visitors and Temporary Residents to the United States

Health requirements that pertain to applicants for an immigrant–permanent resident visa do not apply to visitors and temporary residents. The current US immigration laws require applicants for an immigrant visa to have mandatory medical screening for some infectious diseases and to have up-to-date, age-dependent vaccination coverage before an immigration visa will be issued. Visitors and temporary residents do not receive medical screening for infections such as tuberculosis (TB) and are not required to fulfill US vaccination requirements. No surveillance system is in place to identify health problems in this population. Therefore, medical conditions are known only if a person has a reportable disease, for which reporting to health departments is mandatory. Occasionally, case reports or case series published in academic journals shed some light on health issues encountered. We therefore examined these limited reports on vaccination coverage, disease burden, and healthcare-seeking behavior of nonimmigrant travelers.

## Specific Populations of Nonimmigrants to the United States

### Temporary Residents: International Students and Exchange Visitors

Persons who travel to the United States to study may represent a population at higher risk than others for transmission and spread of infectious diseases. Risk is increased because college campuses provide favorable environments or situations for spread of infectious diseases, e.g., close contact (e.g., in classrooms, in dormitories, and at social gatherings), student behavior, and variable immunity among persons from a wide geographic area (*9*).

In 2008, an estimated 1,052,694 active nonimmigrant students and exchange visitors and their families were in the United States; 68% were enrolled in bachelor, masters, or doctoral degree programs (*10*). The 5 countries from which most international students originated were South Korea, India, China, Japan, and Taiwan. In 2008, the states that hosted more than half (51%) of all enrolled international students were California, New York, Texas, Massachusetts, Illinois, and Florida (Map 1 in the Technical Appendix). Despite this large number of international students, no published data are available regarding their vaccination coverage.

Prevalence of TB varies worldwide and may affect nonimmigrant travelers. In a study conducted during 1997–1998 among incoming international students from 70 countries enrolled in a community college in Iowa, 59 (35%) of 171 had a positive tuberculin skin test result (≥10 mm induration). Of those 59, isoniazid therapy was begun by 34, of which 27 successfully completed the prescribed regimen (*11*). The Iowa study suggests that treatment of latent TB infection in visiting student populations is suboptimal and may represent an area in which improved intervention could prevent illness and spread of infection.

### Temporary Residents: Agricultural Workers

Another group of temporary residents comprises agricultural workers and their families. The Department of Homeland Security defines farm workers as agricultural workers, but other agencies consider them farm workers, crop workers, or agricultural workers. For instance, the federal statutes governing migrant health funds define a migrant farm worker as a person who mainly works in agriculture on a seasonal basis and may migrate from farm to farm within a state, among states, or among countries (*12*).

According to the 2005 National Agriculture Workers Survey results, a large percentage (42%) of agriculture workers in 2001–2002 were migrants. Among the migrants, 26% traveled only within the United States and 35% migrated repeatedly to and from a foreign country (*13*). The Survey also reported that 78% of agricultural workers were foreign born, 50% were younger than 31 years of age, 80% were male, 58% were married, and 57% were living apart from their families (*13*). Of the estimated 3 million seasonal agricultural workers in the United States in 2006, 1 million were hired agricultural workers, ≈50% of whom lacked legal authorization to work in the United States (*14*).

In terms of health status, seasonal agricultural workers frequently live in crowded conditions with poor sanitation and may have suboptimal nutrition; each of these factors is associated with spread of infectious disease. The characteristics of this group may predispose them to infection with, and spread of, TB. In 1996, a study among Hispanic migrant agricultural workers in Indiana found that 28.3% of adult and 7.5% of adolescent (11–18 years of age) agricultural workers had a positive tuberculin skin test result, although no active TB cases were identified. The study also found a high rate of chronic respiratory diseases, which may predispose this population to further consequences of superimposed acute or chronic respiratory infections (*15*). This large mobile population has all factors known to be associated with HIV/AIDS and sexually transmitted infections: members are generally young, mostly male, live in poverty, and have limited access to educational opportunities (*12*,*16*).

Agricultural workers may also be disproportionately prone to injuries and exposed to environmental health hazards. One study reported that 6% of male and 4% of female agricultural workers had at least 1 workplace injury during the 12-month period before the interview (*17*). Other studies have shown that direct contact with pesticides is frequently associated with multiple workplace health conditions, such as irritated eyes, headache, blurred vision, dizziness, numbness, tingling, diarrhea, vomiting, and skin irritation (*18**,**19*). Recent research on the mental health of agricultural workers has found that nearly 40% of workers experience depression and 30% experience anxiety (*20*). A cohort study among agricultural workers in Colorado found pesticide poisoning to be significantly associated with depression (*21*).

The California Agricultural Workers Health Survey, conducted in 2000, found that rates of chronic health conditions for agricultural workers were high; e.g., 81% of male and 76% of female agricultural workers were overweight or obese, predisposing them to diabetes and heart disease (*22*). Lack of available health insurance clearly creates barriers to care and substantially limits access to healthcare services, exacerbating disparities (*6*,*7*). Although 23% of seasonal agricultural workers reported having some type of health insurance, only 8% of seasonal workers and 15% of year-round workers reported that their employer offered them insurance for non–work-related illness or injury (*13*). Even workers who have access to health insurance through employee premium share programs frequently do not enroll because they cannot afford the premiums (*13*). Although compelling information indicates the need for action to serve this vulnerable population, more detailed and systematic data collection would assist in crafting better policies, interventions, and educational tools and materials.

### Visitors: Tourists and Business Travelers

Travel of susceptible or infected persons from disease-endemic to disease-nonendemic areas presents an opportunity for transmission of vaccine-preventable diseases in susceptible populations. This risk is especially great when vaccination rates in disease-nonendemic areas are declining or low. Although national vaccination levels are high in the United States, unvaccinated children tend to be clustered geographically or socially, increasing the risk for transmission of vaccine-preventable diseases (*23**,**24*). Every year, ≈17,000 children in the United States receive no vaccine, primarily for religious, personal, or medical reasons (*24*). Most of these children reside in states that allow exemptions to laws mandating vaccinations for children as they enter school (*24*). During 2000–2001, all states allowed vaccination exemptions for medical reasons, 48 for religious reasons, and 12 for philosophical reasons. Of the states that allow exemptions, 6 (California, Texas, New York, Florida, Illinois, and New Jersey) are also the states of residence for 68% of the children of US immigrants (*25*). These states also receive the largest number of nonimmigrant temporary residents (*1*). The proximity of susceptible populations to large numbers of mobile visitors and temporary residents may represent opportunities for potential sustained transmission of vaccine-preventable diseases.

A recent experience in Europe highlights the risk of allowing vaccination rates to decline. In June 2008, the United Kingdom’s Health Protection Agency declared that measles was again endemic there as a result of an 80%–85% decline in measles vaccination coverage among children <2 years of age (*26*). This decline in coverage resulted from an increase in the number of parents who refused to have their children vaccinated. During the same period, Austria, Italy, and Switzerland also reported measles outbreaks (*27**,**28*). As noted earlier, in 2007 the United Kingdom was among the 4 most common countries of citizenship for short-term temporary residents in the United States (*1*). Although measles was declared eliminated in the United States in 2000, during the first 7 months of 2008, the US Centers for Disease Control and Prevention reported measles outbreaks in 15 states (*29*). Of the 131 confirmed cases, 89% were imported from or associated with importation from other countries, particularly from the previously mentioned countries in western Europe; 91% were in persons who were unvaccinated or of unknown vaccination status. Among the 131 cases, 17 were acquired outside the United States, 9 were in US residents who had traveled, and 8 were in visitors and temporary residents. Among the 112 (91%) confirmed measles cases in unvaccinated persons, 63 (66%) of these persons had not been vaccinated because of philosophical or religious beliefs (*29*). The 2008 measles outbreaks demonstrated the risk for transmission of communicable diseases by travelers returning to the United States and by visitors and temporary residents visiting communities where clusters of people have suboptimal vaccine coverage (Map 2 in the Technical Appendix).

Poliomyelitis is another vaccine-preventable disease that has been reintroduced through mobile populations. During 2003–2006, polio was imported by travelers (e.g., refugees, pilgrims, business travelers) to 24 polio-free countries (*30*). In 2005, the Minnesota State Health Department diagnosed vaccine-derived poliovirus infection in 4 children; the infection had been circulating in 4 children in a predominantly unvaccinated religious community (*31*). No source for the infection could be identified, but the original source of this virus was probably a person who had received oral polio vaccine in another country. Neither the index case-patient nor her family members had any history of international travel. This outbreak raised concerns regarding transmission of the virus to other US communities with low vaccination levels (Map 3 in the Technical Appendix).

TB is a major health problem for US residents and visitors who were born in or have lived in Asia, Africa, Latin America, or eastern Europe, where TB remains highly endemic (*32*). In 2007, the overall incidence of TB in the United States was 4.2 cases per 100,000 population; rates were 2.1 per 100,000 population for US-born persons and 20.6 per 100,000 for foreign-born persons. More than half (51.8%) of foreign-born persons with TB were from 4 countries: Mexico (n = 1,846), the Philippines (n = 952), India (n = 619), and Vietnam (n = 568). Of all reported TB cases in the United States during 2007 (n = 13,292), 52% were reported by the 5 most common destination states for immigrants and visitors and temporary residents (California, Florida, Illinois, New York, and Texas) (*1*,*10**,**33*). A study published in 2004 found that 42% (n = 114) of TB culture–positive cases diagnosed by the Tarrant County Health Department in Texas from 1998 through 2000 were in foreign-born persons. Of these, 67 (59%) were permanent residents, 28 (25%) were undocumented, and 19 (17%) were visitors or temporary residents (*34*).

Many persons may visit the United States without seeking pretravel health consultation. Certain areas in the United States have endemic diseases that visitors and temporary residents are not familiar with such as Lyme disease, Rocky Mountain spotted fever, and West Nile virus encephalitis.

## Current Efforts to Improve the Health Status of Temporary Residents

To improve the health of nonimmigrant temporary residents, government agencies and nongovernment advisory groups are making efforts to ensure that certain categories of nonimmigrant visa applicants are aware of and have access to healthcare services while in the United States. The US Department of State requires exchange visitors (J-1 visa category) and their dependents (J-2 visa category) to have their own medical insurance coverage and enlists program sponsors to ensure compliance with requirements (*35*). However, no similar federal guidance exists for other categories of nonimmigrant visa holders.

In March 2008, the American College Health Association (ACHA) updated its guidelines for student health insurance program standards (*36*). According to these guidelines, as a condition of enrollment students must provide evidence of adequate health insurance coverage for themselves and their dependents. Because of concerns about the spread of vaccine-preventable diseases on college campuses, ACHA also updated its recommendations for prematriculation immunizations to be consistent with the recommendations of the Advisory Committee on Immunization Practices (*37*). In addition, to address the shifting epidemiology of TB to foreign-born persons, ACHA updated its TB control guidelines in July 2008. The new guidelines recommend that US colleges and universities screen all incoming students for active or latent TB (*38*). Resources have been developed to help US colleges and universities institute screening and treatment programs (*39*).

More broadly, many health systems, with substantial support from federal agencies, have begun to meet the healthcare needs of farm workers and their families, such as the federally qualified nonprofit community and migrant health centers that provide primary and preventive health services throughout the United States. The Health Resources and Services Administration, through the Bureau of Primary Care, has developed the Health Disparities Collaborative Program to eliminate ethnic health disparities (*12*) through their health centers and mobile clinics that serve remote farms. In addition, a grassroots movement among local clinics and organizations (the Migrant Clinician Network) has developed tools and materials to support clinics and clinicians who work with farm workers (www.migrantclinician.org).

Many states have begun to develop guidance for providing medical and preventive health services to mobile populations (e.g., immigrants, and refugees), especially in states with a high percentage of these populations, such as Minnesota, Massachusetts, Michigan, New York, and California. These states have identified culturally and linguistically appropriate ways to address the health concerns of foreign-born persons. An exemplary program for linguistically and culturally appropriate healthcare education material in multiple media is the Emergency, Community and Health Outreach network (http://newroutes.org/echo).

In addition to government agencies, several academic institutions and many integrated health systems and even community clinics have begun to train future providers in culturally sensitive and geographically informed healthcare. The number of Academic Global Health Centers in the United States has surged; some programs offer training in the special health issues of mobile populations (*40*).

Although many government agencies, nonprofit organizations, academic institutions, integrated health systems, clinics, and individuals are developing innovative programming and materials, all these efforts are in their infancy and generally not well coordinated. To substantially reduce the disparities of care experienced by mobile populations, including visitors and temporary residents, we need improved data collection, surveillance and scientific evaluation, changes in systems to reduce barriers to care, and increased education and advocacy on behalf of these frequently disenfranchised populations.

A relatively simple, concrete step that could be taken is the development of health awareness programs that try to reach visitors and temporary residents before their arrival in the United States. Such programs could acquaint visitors and temporary residents with US health service requirements and regulations and reduce the burden on the public healthcare system. This goal could be achieved by developing innovative communication tools and messages that address the following: access to the public healthcare system, the Advisory Committee on Immunization Practices vaccination recommendations, and the health insurance coverage policies available in the United States. These messages could be disseminated by many organizations, including the US Department of State, the Centers for Disease Control and Prevention, US university clinics, American cultural centers and education offices at US embassies overseas, health and travel insurance companies that provide emergency health insurance coverage to visitors to the US, and the offices of international banks solicited by the US embassies to receive visa processing fees and distribute visa application forms. Ultimately, to better serve visitors and temporary resident populations, particularly vulnerable populations such as farm workers, more equitable care models and evidence-based clinical best practices must be developed and disseminated.

## Conclusions

Nonimmigrant visitors and temporary residents represent a considerable and increasing percentage of travelers to the United States (*1*). Information is limited with regard to the health status of visitors and temporary residents upon arrival and their need for and use of medical services in the United States. More information is needed to determine the public health issues as well as the health challenges and needs of visitors and temporary residents in the United States. After these issues and needs have been clarified, intervention programs should be developed to increase access and decrease the disparities of care experienced by these populations.

## Supplementary Material

Technical Appendix Health Status of Visitors and Temporary Residents, United States
