# Supplementary material for: Health Status of Visitors and Temporary Residents, United States
Source: Emerg Infect Dis. 2009 Nov;15(11):1715–20. doi: 10.3201/eid1511.090938 (PMC2857256; doi:10.3201/eid1511.090938)
Supplement: Technical Appendix — Health Status of Visitors and Temporary Residents, United States [file 09-0938_Techapp-s1.pdf]

Health Status of Visitors and Temporary Residents, United States

Technical Appendix

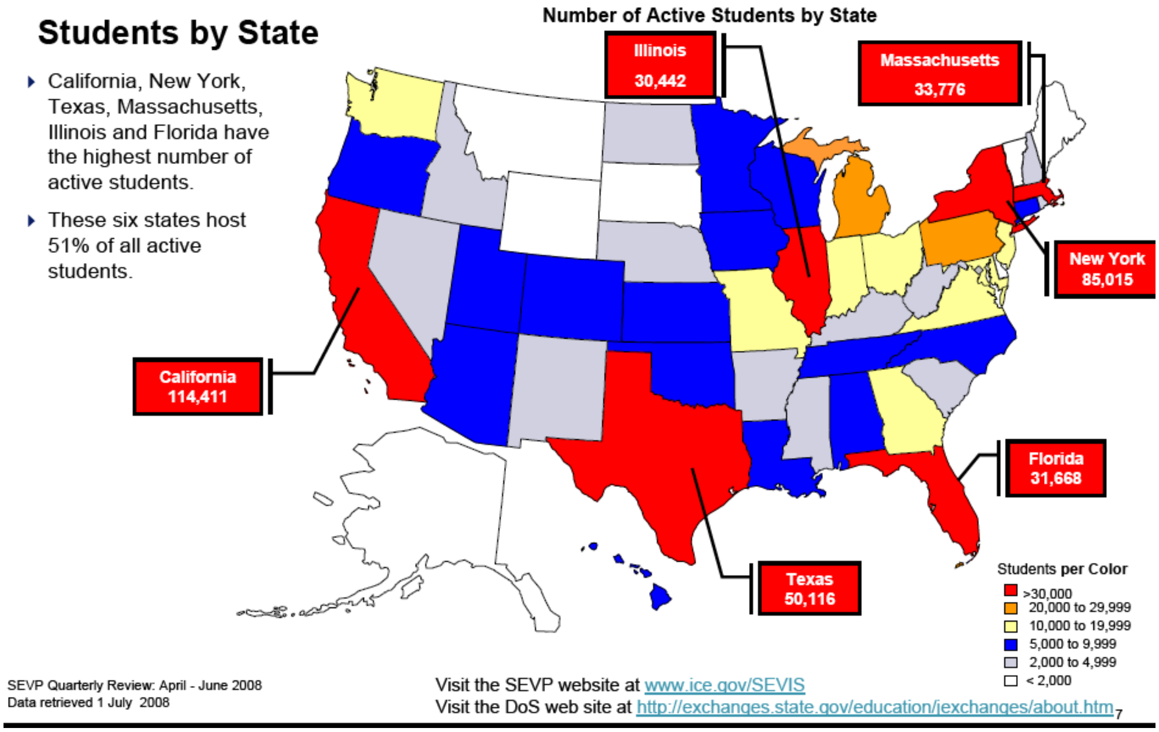

Map 1. Number of students and exchange visitors, by state, United States, 2008. Source: the Student and Exchange Visitor Program (SEVP) website, www.ice.gov/SEVIS, accessed July 2, 2008

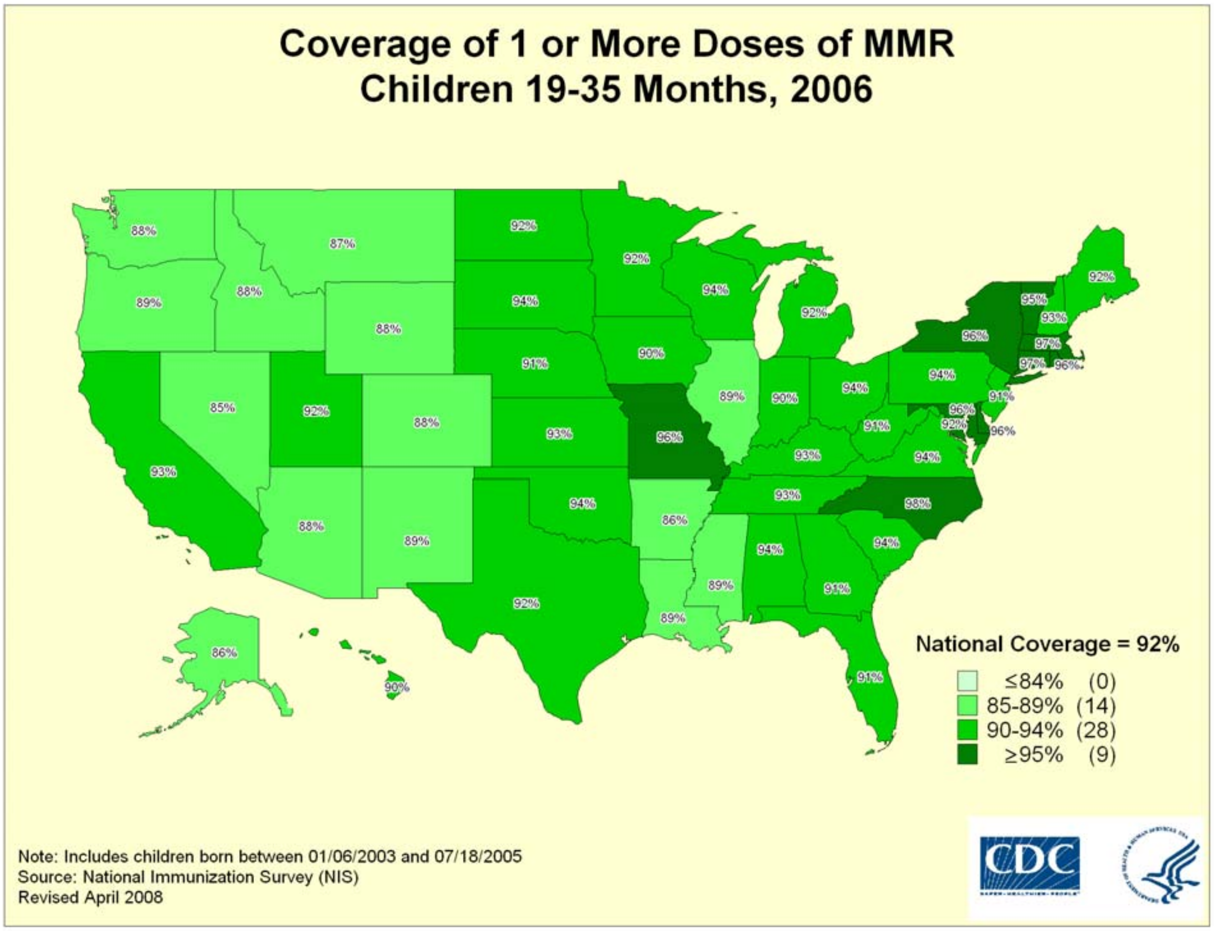

Map 2. Coverage of ≥1 doses of measles, mumps, and rubella (MMR) vaccine in children 19–35 months of age, United States, 2006.

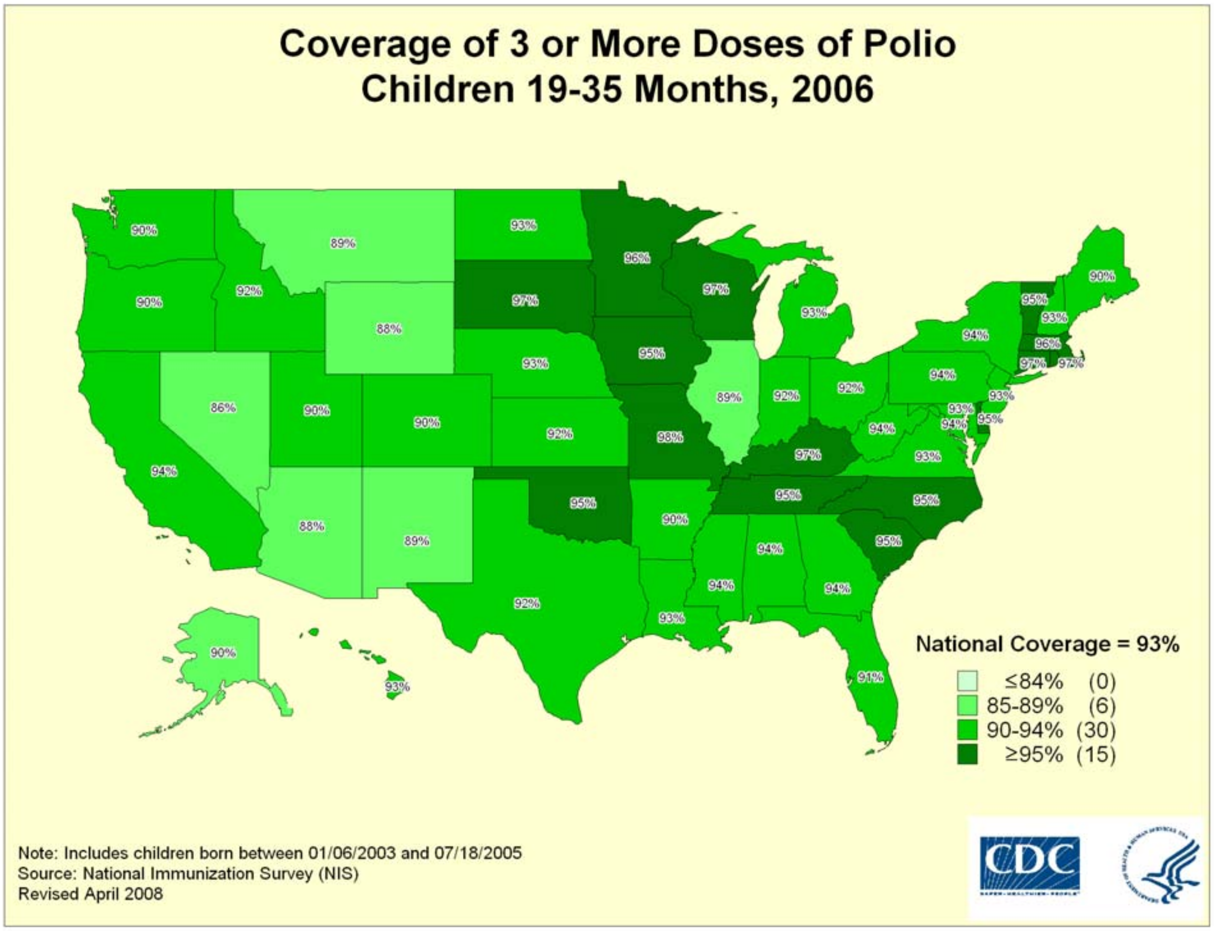

Map 3. Coverage of ≥3 doses of polio vaccine in children 19–35 months of age, United States, 2006.
